# An mHealth Intervention to Address Depression and Improve Antiretroviral Therapy Adherence Among Youths Living With HIV in Uganda: Protocol for a Pilot Randomized Controlled Trial

**DOI:** 10.2196/54635

**Published:** 2024-03-08

**Authors:** Proscovia Nabunya, Patricia Cavazos-Rehg, James Mugisha, Erin Kasson, Olive Imelda Namuyaba, Claire Najjuuko, Edward Nsubuga, Lindsey M Filiatreau, Abel Mwebembezi, Fred M Ssewamala

**Affiliations:** 1 International Center for Child Health and Development Brown School Washington University in St. Louis St. Louis, MO United States; 2 Department of Psychiatry School of Medicine Washington University in St. Louis St. Louis, MO United States; 3 College of Health Sciences Makerere University Kampala Uganda; 4 International Center for Child Health and Development Field Office Masaka Uganda; 5 Division of Computational & Data Sciences McKelvey School of Engineering Washington University in St. Louis St. Louis, MO United States; 6 Division of Infectious Diseases School of Medicine Washington University in St. Louis St. Louis, MO United States; 7 Reach the Youth Kampala Uganda

**Keywords:** depression, adherence, mHealth, cognitive behavioral therapy, antiretroviral therapy, youth living with HIV, Uganda

## Abstract

**Background:**

People living with HIV often struggle with mental health comorbidities that lower their antiretroviral therapy (ART) adherence. There is growing evidence that depression treatment may improve ART adherence and result in improved HIV outcomes. Given that mental health services are severely underequipped in low-resource settings, including in Uganda, new solutions to increase access to mental health care and close the treatment gap are urgently needed. This protocol paper presents the Suubi-Mhealth study, which proposed to develop a mobile health (mHealth) intervention for use among Ugandan youths (14-17 years) with comorbid HIV and depression, taking into account their unique contextual, cultural, and developmental needs.

**Objective:**

The proposed study is guided by the following objectives: (1) to develop and iteratively refine an intervention protocol for Suubi-Mhealth based on formative work to understand the needs of youths living with HIV; (2) to explore the feasibility and acceptability of Suubi-Mhealth on a small scale to inform subsequent refinement; (3) to test the preliminary impact of Suubi-Mhealth versus a waitlist control group on youths’ outcomes, including depression and treatment adherence; and (4) to examine barriers and facilitators for integrating Suubi-Mhealth into health care settings.

**Methods:**

Youths will be eligible to participate in the study if they are (1) 14-17 years of age, (2) HIV-positive and aware of their status, (3) receiving care and ART from one of the participating clinics, and (4) living within a family. The study will be conducted in 2 phases. In phase 1, we will conduct focus group discussions with youths and health care providers, for feedback on the proposed intervention content and methods, and explore the feasibility and acceptability of the intervention. In phase II, we will pilot-test the preliminary impact of the intervention on reducing depression and improving ART adherence. Assessments will be conducted at baseline, 1-, 2-, and 6-months post intervention completion.

**Results:**

Participant recruitment for phase 1 is completed. Youths and health care providers participated in focus group discussions to share their feedback on the proposed Suubi-Mhealth intervention content, methods, design, and format. Transcription and translation of focus group discussions have been completed. The team is currently developing Suubi-Mhealth content based on participants’ feedback.

**Conclusions:**

This study will lay important groundwork for several initiatives at the intersection of digital therapeutics, HIV treatment, and mental health, especially among sub-Saharan African youths, as they transition through adolescence and into adult HIV care settings.

**Trial Registration:**

ClinicalTrials.gov NCT05965245; https://clinicaltrials.gov/study/NCT05965245

**International Registered Report Identifier (IRRID):**

DERR1-10.2196/54635

## Introduction

### Overview

Globally, an estimated 39 million people were living with HIV in 2022; of these, 1.5 million were children <15 years [1]. Approximately 84% of new HIV infections among children <15 years occurred in sub-Saharan Africa (SSA) [1]. HIV disproportionately impacts children from poor and disadvantaged backgrounds, and the prevalence of HIV–related complications and mortality are highest among those struggling with poverty and deprivation [2,3]. Poor children living with HIV are more likely to experience compromised health, inconsistent antiretroviral therapy (ART) adherence, and elevated mental health difficulties, including depression, which increases the risk of HIV transmission [2,3]. In Uganda, the focus of this study and one of the SSA countries hardest hit by HIV, over 80,000 children (0-14 years) are living with HIV [4]. Improvements in access and use of HIV services, including testing, care, and availability of free ART have reduced child mortality [5] and resulted in an increased number of children now growing up with a highly stigmatizing and transmittable infection.

Depression is the most common psychiatric disorder among people living with HIV [6,7]. The prevalence of depression among people living with HIV is estimated to be as high as 63% in SSA [8]. Among young people, the prevalence of major depression is estimated between 16% and 40.8% [9]. Depression is associated with compounding and exacerbating negative HIV outcomes [10,11]. While high levels of ART adherence are necessary for young people to benefit individually from ART, as well as for reducing the risk of HIV transmission, depression has been found to significantly impede ART adherence [10,11]. Previous studies have found that patients with depression are nearly 3 times more likely to be nonadherent to medication regimens than patients without depression [12,13]. Depression can impact ART adherence through isolation from social support, impaired adaptive coping skills, apathy, forgetfulness, and hopelessness [14]. In addition, depression-related fatigue, low energy, inconsistent or lack of sleep, and loss of appetite may make it hard to take ART and attend medical appointments [15]. Indeed, due to limited ART adherence and associated medical complications, patients with depression have a higher risk of increased HIV viral load [16], rapid disease progression, and mortality relative to patients who are not depressed [17,18], making this a crucial population with which to intervene. Thus, failure to address the mental health needs of youths living with HIV, including depression, may lead to costly long-term consequences.

There is growing evidence that depression treatment may improve ART adherence and result in improved HIV outcomes [19,20]. Studies have shown that treating depression was associated with up to 83% higher odds of adhering to HIV treatment [19,21]. Among adults, improvements in cognitive processes have been shown to increase confidence and motivation to engage in HIV management behaviors [22,23]. However, few studies targeting the treatment of depression and evaluating the subsequent improvement in ART adherence have been conducted with adolescents [24], and even fewer studies have been conducted with populations from SSA [25]. Therefore, it is crucial to develop interventions for depression that are tailored to the needs of this specific population, given the prevalence of HIV in the region and the elevated risk factors and myriad barriers to care.

Youths are the lowest ART adherent age group and are at the highest risk of dropping out of ART care programs [26-28]. Poor ART adherence among youths is due, in part, to their transition from youth-focused care into adult-focused care that is less accommodating to the needs of this age group who are at the cusp of taking on new roles and responsibilities including their own disease management [29-31]. Moreover, insufficient staff training to support youths living with HIV during this period, lack of pediatric ART formulations in adult clinics, insufficient clinical monitoring, rigid scheduling that interferes with schooling, as well as loss of pediatric clinic relations with peers and clinical support staff all contribute to poor engagement in care during this transition period [27,28]. Indeed, studies indicate that this period in the life course is associated with a significant drop in ART adherence with up to 50% of youths discontinuing their HIV care entirely during this shift [32]. Thus, targeted interventions to improve retention and treatment outcomes during this period are critical.

Cognitive behavioral therapy (CBT) is a promising approach to reduce depression and improve ART adherence [33-35]. CBT is a form of psychotherapy that is focused on changing patterns of thinking and the associated behaviors [36]. CBT has a growing evidence base as an efficacious approach to reducing depressive symptoms and improving ART adherence among people living with HIV [24,33,37-39]. Moreover, evidence supports the efficacy and effectiveness of CBT in reducing self-stigma for people with mental illness—one of the major barriers to treatment adherence [40,41]. Some key principles of CBT that specifically target ART adherence and depression include instruction for behavioral activation, cognitive restructuring that addresses negative automatic thoughts, problem-solving, and relaxation training [39,42,43]. Despite the promise of CBT, youths with depression living with HIV, particularly those in resource-constrained settings such as Uganda, are challenged by numerous barriers to treatment including lack of access to health care and a severe shortage of trained providers [43-45]. As such, there is a need for interventions developed to take into account these numerous treatment barriers and to structure intervention components and delivery methods to fill the gaps in the current system of traditional assessment and treatment access.

A mobile health (mHealth) intervention is a feasible approach for the delivery of interventions to youths with depression living with HIV in Uganda. Upwards of 85% of countries in SSA have accomplished a high level of mobile phone penetration [46]. According to the 2021 Uganda Communications Commission Report, over 70% of Ugandans own a mobile phone [47]. Indeed, several studies in Uganda have used mobile technology to deliver health interventions, including the promotion of correct HIV or AIDS knowledge and testing [48], improving patient-provider communication [49], increasing clinic attendance among individuals on ART [50], and improving HIV care [51].

However, none have focused on addressing depression among youths living with HIV. Moreover, in our own work in Uganda, 80% of youth indicate access to a mobile phone [52]. In neighboring Kenya, a mobile app tested to deliver individual counseling services and facilitate peer support among youth living with HIV documented positive experiences, peer network development, as well as benefits related to treatment adherence, stigma reduction, and mental and behavioral health [53]. Thus, given the many barriers to care experienced by Ugandan youths living with HIV, as well as the lack of access to trained providers [54,55], the proposed Suubi-Mhealth intervention is a viable and sustainable approach for treating depression treatment in this priority population.

This Suubi-Mhealth study will develop a mHealth intervention for use among Ugandan youth with comorbid HIV and depression, taking into account their unique contextual, cultural, and developmental needs. This mobile app will apply user-centered design methodologies we have used to develop and evaluate similar evidence-based digital therapies [56]. The study will be conducted in 2 phases and will be guided by the following specific aims:

1. Phase 1, aim 1: develop and iteratively refine an intervention protocol for Suubi-Mhealth based on formative work to understand the needs of youths with depression living with HIV (14-17 years).
2. Phase 1, aim 2: based on the results of aim 1, we will explore the feasibility and acceptability of Suubi-Mhealth for use with youths with depression living with HIV on a small scale to inform subsequent refinement for the larger phase of this project.
3. Phase 2, aim 1: pilot-test the preliminary impact of Suubi-Mhealth versus a waitlist control group, on reducing depression (primary outcome) and improving ART adherence, mental health functioning, quality of life, and lowering HIV stigma (secondary outcomes).
4. Phase 2, aim 2: qualitatively examine barriers and facilitators for integrating Suubi-Mhealth into health care settings.

### Theoretical Frameworks Guiding the Study

The Suubi-Mhealth study is guided by two complementary frameworks: (1) the mHealth development and evaluation framework [57], and (2) PRISM (Practical, Robust Implementation, and Sustainability Model) [58]. The mHealth development and evaluation framework involves an iterative process for refining a mHealth intervention through integrating various information sources including published evidence, theory, and formative research with the target group [57]. Specifically, this process involves the following steps: (1) formative research to inform the development of the intervention content and regimen; (2) pretesting to determine the acceptability of the proposed intervention, improve and refine the intervention based on feedback; (3) pilot study to test intervention content and regimen as well as the process, including recruitment and data collection; (4) randomized control trial to test the effect of the intervention in comparison with a control group; (5) qualitative research to improve the intervention and implementation issues and methods; and (6) evaluation and implementation to determine the effect of the intervention once scaled up [57].

Similarly, PRISM is a comprehensive intervention development and implementation framework [58]. It emphasizes the following: (1) organizational perspectives on an intervention (eg, feasibility, adaptability, and barriers); (2) external environment (eg, community resources); (3) recipients’ characteristics (youths, provider, and parent response); and (4) implementation and sustainability infrastructure (training and supervision supports). PRISM provides a framework to study the interaction of interventions with the characteristics of multilevel contexts or factors that may influence uptake, implementation, integration, and youths’ outcomes (youths’ responses, provider preparedness, motivation and fidelity, and community-level support).

## Methods

### Study Overview

**Figure 1:**
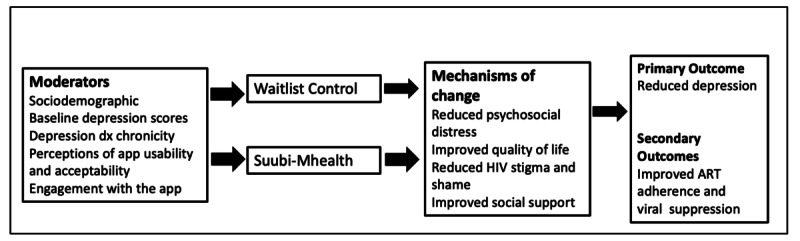
Suubi-Mhealth conceptual model. ART: antiretroviral therapy.

The overall goal of this study is to develop a mHealth intervention (Suubi-Mhealth) for use among Ugandan youths with comorbid HIV and depression, taking into account their unique contextual, cultural, and developmental needs. In phase 1 of the study, we will conduct 4 focus group discussions, each with 6-8 youths and 2 focus group discussions with health care providers, for feedback on the proposed intervention content and methods. Based on the results from focus group discussions, we will recruit 30 youths to engage with the Suubi-Mhealth app to explore its feasibility and acceptability so as to inform subsequent refinement for the larger phase of this project. In phase 2, a total of 200 youth from 10 health clinics will be recruited, and randomly assigned to either the Suubi-Mhealth condition or waitlist control condition (5 clinics with 100 youths per condition), to pilot-test the preliminary impact of the intervention on reducing depression and improving ART adherence, mental health functioning, quality of life, and lowering HIV stigma. Upon intervention condition, we will qualitatively examine participants’ intervention experiences, as well as barriers and facilitators for integrating Suubi-Mhealth into health care settings. The hypothesized relationships are provided in Figure 1 below.

### Research Setting

The Suubi-Mhealth study will be implemented in the greater Masaka region located in Southern Uganda, with one of the highest HIV prevalence in the country [4]. Youths will be recruited from health clinics where the International Center for Child Health and Development (ICHAD), which will house the study, and our collaborating institution, Reach the Youth Uganda, operate. For phase 1, youths will be selected from 2 clinics within 1 district. These clinics will be excluded from phase 2. For phase 2, we will select 10 clinics based on size (total number of youth served) and health facility level (levels II, III, IV, or hospital) in each district. For a health clinic to be included in the study, it will have to be credited by the Uganda Ministry of Health to provide ART and have adolescent-friendly services, for example, adolescent clinic days. The procedures have been used in our other studies implemented in the study region [52,59,60].

### Inclusion Criteria

Youths will be eligible to participate if they meet the following criteria: (1) 14-17 years of age with the cognitive ability to understand and comprehend the assenting process; (2) HIV-positive and aware of their status, that is, disclosed to; (3) receiving ART and care from one of the participating clinics; and (4) living within a family, including with extended family members (not in institutions). We will identify youths with depressive symptoms by administering the Patient Health Questionnaire-9 (PHQ-9) [61], which has been validated in rural settings in Uganda [62,63]. Youth scoring ≥10 on the PHQ-9 will be considered for enrollment. Youths will be excluded if they do not meet the inclusion criteria above or are unable to understand the informed consent process, or inability or unwillingness to commit to study completion. Health care providers will be recruited if they are working directly with youths living with HIV at their clinics and agree to participate in the study.

### Participant Recruitment and Consent

Procedures tested in our previous and ongoing studies in the study region will be used [59,60,64,65]. Specifically, participants will be identified and recruited from health clinics that are currently collaborating with ICHAD and Reach the Youth in the study region. A list of eligible participants will be created from medical records. Health care providers will present the study to adult caregivers of eligible youth during adolescent clinic days. These are scheduled days when youths visit clinics for ART refills, counseling, and treatment check-ins. If caregivers are interested, verbal consent to be contacted by a research staff (who will be onsite) will be requested. Next, the adult caregiver and the youth will separately provide consent or assent for the youth to be screened. Youths who meet the inclusion criteria and their caregivers (separately) will undergo an informed consent process to participate in the study. The consent and assent forms will clearly state that the youth can withdraw from the study at any time, for any reason, with no explanation, and will not be penalized. Caregivers will sign the consent form consenting for their children to participate. Youths will sign an assent form. If either the youth or the caregiver refuses to participate, the youth will not be enrolled. Health care providers will go through the same process and sign a standard consent form.

### Sample Tacking and Retention

The project will take place in a highly stable region of Uganda, where geographical moves are rare. In addition to recommendations garnered by focus group discussion participants in aim 1, we will use several practices to increase retention. During our interviews, youths will be asked to provide details of contacts, including telephone number, and names, addresses, and contact information for 3 people who will always know how to reach them. Participants will be reminded that if the research team were to contact the people listed, the team would never discuss any details about the participant’s involvement in the study. A phone call reminder will be made prior to each assessment. Our team has effectively used these methods in all our research projects, resulting in very low attrition [66-69]. We will keep careful records for those who drop out of the study and test for attrition bias based on data collected prior to study dropout. To the extent that such bias is present, we will limit generalizations accordingly, or, where possible, introduce statistical adjustments to address bias. Retention efforts will be periodically reassessed, reviewing best practices to reduce attrition.

### Ethics Approval

We obtained approval for all study procedures from the Uganda Virus Research Institute Research Ethics Committee (Ref: GC/127/919) on August 29, 2022, Washington University in St. Louis Institutional Review Board (IRB; 202209115) on October 06, 2022, and from the Uganda National Council for Science and Technology (Ref: SS1442ES) on November 15, 2022. The study is registered in the Clinical trials.gov database (Identifier: NCT05965245).

### Description of the Study Conditions

In phase 2 of the study, participants will be randomized to 2 conditions (100 youths per condition): a waitlist control condition or the Suubi-Mhealth condition, as detailed below.

#### Waitlist Control Condition

A waitlist control condition method will be used [70]. Participants will be randomly assigned to a waitlist and will receive the Suubi-Mhealth intervention after the active treatment group. In addition, participants will receive a smartphone without the Suubi-Mhealth app at the same time as the intervention group.

#### Suubi-Mhealth Treatment Condition

##### Overview

Participants in this condition will receive the Suubi-Mhealth intervention, with the specific components described below. All modules will be available in text, video, and audio format. All app content will be available in Luganda local language.

##### Evidence-Based Multimedia Psychoeducational Content to Reinforce Treatment Plans

Educational content will be provided within Suubi-Mhealth and broken into 20 modules. Users will be provided access for 2 months and will be instructed to review ≥2 modules per week. The research team has aligned Suubi-Mhealth content with state-of-the-science recommendations for CBT for ART adherence and depression as applied to HIV medication adherence [33]. In addition, the research team has implemented a group- CBT intervention to address depression and HIV-related stigma among adolescents [59,71]. This manualized intervention, developed with close consultations with mental health experts in the region, will be adapted into Suubi-Mhealth content. Core instruction will include psychoeducation on the interplay between HIV and depression; minimizing cognitive distortions related to ART adherence, stigma, and depression; challenging negative automatic thoughts; analysis and development of behavioral skills; reducing environmental stressors; creating support and helping communicate concerns adaptively and clearly; and problem-solving [37,38].

##### Safety Plan

To ensure safety while using the app, participants will be informed that the social networking center is informational only and not equipped for managing crises. Messages from users will be reviewed by a trained member of the research team within 24 hours. If signs of an imminent crisis are detected, a supervisor will send a crisis referral, make a report, and notify the parent so that no user waits longer than 24 hours to receive support. Emergency contact information will be shown in the corner of the survey page for all assessments.

##### Goal Setting and Self-Monitoring Features to Enable Self-Management

Our previous mHealth participants have indicated their desire for a goal-setting feature within an app, and studies of mobile apps have demonstrated that goal-setting, self-tracking, and monitoring features are essential to facilitate self-management skills and sustain user engagement [72,73]. With this in mind, there will be an ability to set goals and monitor symptoms of depression over time within Suubi-Mhealth. Rewards within an app have been shown to motivate and sustain healthy behaviors [74]. Our rewards feature will be used to inspire completing psychoeducation content and attaining goals by awarding digital “trophies” for participants who successfully meet a goal or reach a milestone (eg, complete a module on how to challenge negative automatic thoughts).

##### Clinical Dashboard to Monitor Participants’ Use of Suubi-Mhealth

A clinical management dashboard will make it efficient for the research team to input participant data and monitor participant use of the app and assessment responses.

### Data Collection

Data will be obtained through an eligibility screening checklist, computer-assisted, interviewer-administered structured questionnaires, focus group discussions, and semistructured in-depth interviews. All assessments will take place at the clinic, participant’s home, or ICHAD’s field offices in Masaka. Assessments will be conducted by trained Ugandan Research Assistants.

### Qualitative Assessments

Focus group discussions and semistructured interviews will be conducted. In phase 1, we will conduct 4 separate 60-minute in-person focus group discussions (6-8 youths each) and 2 focus group discussions for health care providers. Participants will be asked about impressions of expert-suggested content and design and their own suggested components for Suubi-Mhealth that could be of greatest utility for unique needs. They will also be asked for input regarding methods to increase participation and retention for follow-up assessments. All focus groups will be conducted in Luganda, will last approximately 1-hour and will be held in a private location at each clinic. In addition, we will conduct a 30-minute semistructured qualitative interview with each participant to further explore their experiences with Suubi-Mhealth, including perceived benefits and challenges of participation, and suggested modifications to improve specific components of the intervention. Complications or technical glitches with the app, and how it fared in regard to its ease of understanding, helpfulness, intuitive flow, presentation, and pacing will also be assessed. We will also conduct in-depth interviews with research staff who will be moderating the app.

In phase 2, we will conduct semistructured in-depth interviews with 30 participants upon completion of the intervention to explore their experiences with the intervention as well as barriers and facilitators to implementation and participation. Participants will be purposefully sampled [75], using a combination of high- and low-app users, that is, those in the upper and lower quartiles of engagement with the app (15 participants per group), to take part in a 30-minute semistructured qualitative interview. Interviews will focus on participants’ experience with Suubi-Mhealth and how it fared in regard to its ease of understanding, helpfulness for supporting ART adherence and depression, intuitive flow, presentation, pacing, and the likelihood that they would recommend this intervention to a peer. Reactions to specific content and suggestions for improvement will also be assessed. We will additionally perform in vivo observations in which participants actively use the tool during the interviews to more directly observe challenges to use and implementation.

### Quantitative Assessments

Quantitative assessments will be administered as follows. In phase 1, interviewer-administered quantitative assessments will be collected at 2 months post intervention to examine the usability characteristics and engagement with Suubi-Mhealth. To assess usability characteristics, we will administer a modified version of the 19-item USE (Usefulness, Satisfaction, and Ease of Use) questionnaire to measure usability characteristics of Suubi-Mhealth [76,77]. The USE will ask participants to rate the ease of use and learning (ie, efficiency), usefulness (ie, technical effectiveness), and likability (ie, satisfaction) of Suubi-Mhealth on a 7-point Likert scale (with 1= strongly disagree and 7= strongly agree).

To assess engagement with Suubi-Mhealth, automatically captured data within the app platform will be used to measure app engagement for each participant, including (1) the number of modules completed, (2) the number of messages sent, (3) the number of times self-monitoring and goal-setting features used, (4) the number of times logged into the app, (5) the time spent in the app once logged in, and (6) the time difference between app log-ins.

In phase 2, assessments will be completed at baseline and at 1, 2, and 6 months post intervention to evaluate whether participants’ ART adherence and self-reports on symptoms of depression, mental health functioning and physical health, and quality of life and stigma improve with access to Suubi-Mhealth and whether improvements remain consistent over time. The measures to be used are provided in Table 1 below.

**Table 1:** Phase 2 assessment measures.

Variable	Measures
Demographics	Sociodemographic questionnaire
Depressive symptoms	Patient Health Questionnaire-9 (PHQ-9) [61] and Brief Symptom Index (BSI) [78]
Physical health	The Medical Outcomes Study HIV Health Survey (MOS-HIV) [79]
Quality of life	Pediatric Quality of Life Inventory (PedsQL 4.0) [80,81]
HIV shame	Shame questionnaire [82,83]
HIV stigma	Berger HIV Stigma Scale [84]
Social support	The Multidimensional Scale of Perceived Social Support [85] and Social Support Behavior Scale (SS-B) [86]
ART^a^ adherence	Viral load; self-reports [87]
Intervention feedback	In-depth interviews

^a^ART: antiretroviral therapy.

### Data Analysis Procedures and Milestones

#### Phase 1, Aim 1: to Develop and Iteratively Refine an Intervention Protocol for Suubi-Mhealth Based on Formative Work

##### Overview

We will conduct 4 focus group discussions, each with 6-8 youths (N=32), and 2 groups with health providers (n=16) for feedback on proposed intervention content and methods to increase participation and retention. Focus groups will be audio-recorded and transcribed verbatim. Transcripts will be translated into English and imported into Dedoose to assist with qualitative data management and coding. Inductive thematic analysis of focus group transcripts will be conducted by 2 research team members using the 6 steps described by Braun and Clarke [88] (getting familiar with the data, creating initial codes, looking for themes, reviewing and refining themes, defining and naming themes, and producing the report).

##### Milestone for Aim 1

We will have garnered input from youths and health care providers about the proposed Suubi-Mhealth content, features, and design. Participants will provide feedback on additional suggested components for Suubi-Mhealth that could be of the greatest utility for their unique needs. Suubi-Mhealth will be refined accordingly including its technical aspects, features to add or edit, and content updates or revisions. Participants will also be asked for input regarding methods to increase participation and retention for follow-up assessments. The study design and methods will incorporate feedback received from participants. Specifically, the research team will incorporate relevant suggested content and features into the Suubi-Mhealth intervention protocol before testing.

#### Phase 1, Aim 2: to Explore the Usability, Feasibility, and Acceptability of Suubi-Mhealth in a Small Sample to Inform Subsequent Refinement for the Larger Phase

##### Overview

Following the integration of input and feedback from focus groups to update and refine Suubi-Mhealth (phase 1, aim 1), we will β test Suubi-Mhealth among 30 youths. This is within the range of sample sizes for other pilot tests of digital therapeutics [77,89], and the suggested number of participants for pilot studies [90]. This sample size should be sufficient to explore the usability of and engagement with Suubi-Mhealth and for data saturation for qualitative interviews [91]. Participants will be instructed to use all features of Suubi-Mhealth for 2 months for the purpose of this β test. Two months for β testing is similar in length to other pilot tests for mental health apps [92,93], and will allow sufficient time for participants to engage with as many of the modules and features as they would like. Upon completion, we will garner feedback from each participant via mixed methods exploratory design in order to inform further enhancements of Suubi-Mhealth prior to the RCT (phase 2)*.* We will conduct both quantitative assessments to examine the utility and usability of the Suubi-Mhealth and qualitative interviews to explore participants’ experiences with the app.

##### Analysis Procedures

Quantitative assessments will be recorded using Qualtrics, an electronic data capture system. The data will be exported into the Statistical Analysis System for analysis. App usability will be examined using descriptive measures (mean, SD, median, and range) on the efficiency, technical effectiveness, and satisfaction components of the USE questionnaire. Descriptive statistics will also be used to describe engagement with the app (eg, the median number of logins and time spent in the app). Qualitative data will be analyzed in Dedoose following the same procedures provided in aim 1 above.

##### Adaptation and Feedback Integration Process

We will use procedures similar to those in our other studies for adapting mental health interventions based on target user feedback [94]. Specifically, participants’ feedback gathered from content and usability testing, as well as from experiences obtained during in-depth interviews, will be incorporated into the app based on the level of importance and feasibility including cost. The research team, including the research staff overseeing the app and selected youth, will go over each module to make sure that relevant revisions have been incorporated. Together, the research team will review and approve the adapted content and design before launching phase 2.

##### Milestone for Aim 2

Participants will have, on average, engaged with 75% or more of the mobile app modules and will express enthusiasm over its potential to be a supportive tool. Participants will score Suubi-Mhealth as having high ease of use and learning (ie, efficiency), usefulness (ie, technical effectiveness), and likability (ie, satisfaction). Participants’ provided feedback will be integrated into the app design.

#### Phase 2, Aim 1: to Pilot-Test the Preliminary Impact of Suubi-Mhealth

##### Overview

During phase 2, participants will be randomized to the intervention condition to engage with the Suubi-Mhealth app or the control condition (waitlist control group) to receive the app after the active intervention period. Assessments will be completed at baseline and at 1 month, 2 months, and 6 months post intervention to evaluate whether participants’ self-reports on symptoms of depression, ART adherence, mental health functioning, quality of life, and stigma improve with access to Suubi-Mhealth and whether improvements remain consistent over time.

Participants will also complete the USE measure to assess acceptability and usability, as well as in-depth interviews to explore their experiences with the intervention.

##### Analysis Procedures

Following guidelines for clinical trial analysis, all primary analyses will be intention-to-treat analyses and missing data will be handled using multiple imputations.

##### Mixed-Effect Models

The principal strategy to examine primary and secondary outcomes over time between the Suubi-Mhealth group and the control group will be the use of linear mixed models and generalized linear mixed models, including a random intercept to allow for the correlation of within-participant measurements over time. Models will include the fixed effects of the intervention group (Suubi-Mhealth vs control), time, and group-by-time interaction. Effect sizes and 95% CIs will be reported.

##### Moderation Analysis

First, we will provide descriptive statistics on engagement (session duration, number of modules viewed) and usability scores. We will then use moderation analysis to examine whether Suubi-Mhealth engagement and usability scores moderate the effect of the interventions on our primary and secondary outcomes of interest (ie, depression, ART adherence, psychological distress, overall quality of life, shame, and HIV stigma).

##### Structural Equation Models

We will use mediation analysis to examine whether depression symptoms mediate ART adherence. We will test for mediation using structural equation models with CIs derived from bootstrapping of indirect and total effects. By applying mediation analyses, we will be simultaneously testing whether the intervention engages the targets and whether intervention-induced changes in targets are associated with clinical benefit. Models will account for baseline scores. For sensitivity analyses, we will use a per-protocol approach to address noncompliance by only including participants who completed the study according to the protocol.

##### App Engagement and Usability

Basic descriptive statistics will be reported for Suubi-Mhealth engagement (session duration and number of modules viewed), as well as app usability scores (USE scores and subscales of efficiency, technical effectiveness, and likability). To determine the relationships between app usability and engagement, we will use linear regression models with an index of engagement (derived from principal components analysis using the above raw metrics of engagement) as the dependent variable and overall usability score as the primary predictor of interest. Demographics associated with both the usability scores and the index of engagement will be included as covariates in the model. In a separate model, we will include each usability subscale (efficiency, technical effectiveness, and satisfaction) rather than the overall usability score in order to explore which subscale might be most predictive of engagement with the mobile app-based tool. In addition, linear mixed models will be used to investigate the association between the intervention engagement index with the primary outcomes over time. Covariance structures for the models will be selected as described above. Effect sizes and 95% CIs will be reported.

##### Sample Size and Power

The primary outcome in aim 1 is whether depression decreases between baseline and postintervention initiation. This is calculated from the depression score change between these 2 time points and categorized as yes versus no. The intracluster correlation for clinics is assumed to be 0.093 and the rate of depression decrease in the control group is 5% [95]. Given our total sample (N=200, 100 youths per condition, 5 clinics per condition), we expect to achieve at least 80% power to detect a rate difference of depression decrease when the rate of depression decrease in the intervention group is 40% and the 2-sided significance level of the test is .05.

##### Milestone for Phase 2, Aim 1

Successful completion of a parallel-group RCT to test the feasibility, acceptability, and preliminary efficacy of Suubi-Mhealth*.* Participants will not experience any adverse events. Participants will have engaged with Suubi-Mhealth daily for 2 months and engaged with at least 75% or more of the modules.

#### Phase 2, Aim 2: to Examine the Barriers and Facilitators for Integrating Suubi-Mhealth Into Real-World Contexts

##### Overview

Upon completion of the intervention, we will conduct semistructured in-depth interviews with 30 participants upon completion of the intervention to explore their experiences with the intervention as well as barriers and facilitators to implementation and participation. Based on prior research [79], we anticipate that barrier themes may include (1) lack of knowledge and self-efficacy in using mHealth tools; (2) participants’ inability to receive mobile service due to lack of service coverage and connectivity issues; (3) participants’ inability to understand, maintain, or properly use the mHealth tool due to technology literacy issues; and (4) financial sustainability concerns related to the inability to charge a fee for service, bill, or otherwise be reimbursed for services provided via technology. We anticipate that facilitators may include working collaboratively with the app development team to address technical barriers and adapt the app to meet participants’ needs and consistently reviewing app use data to inform progress [80]. The perceived relative advantage of the app over usual care, the ease of use and ability to adapt the app to improve participants’ use, and awareness of participants’ needs and resources will also likely serve as facilitators.

##### Milestone for Phase 2, Aim 2

Completion of an implementation evaluation in which barriers and facilitators are successfully identified as actionable inputs to inform the integration of Suubi-Mhealth into real-world contexts for youths with depression living with HIV. To ensure that this assessment is constructive in facilitating future implementation, we will map each key barrier and facilitator to a relevant implementation strategy identified in the literature to address a similar barrier or leverage a similar facilitator for maximal benefit [96,97]. For example, if the barriers related to service coverage and connectivity issues are mentioned, then the actionable solution will include working with a different service or network provider. This process will yield a concise yet informative implementation toolkit to support future implementation efforts that may face similar patient, provider, and organization-level challenges.

#### Data Integration and Triangulation

Findings from qualitative and quantitative data analyses will be integrated during the interpretation and discussion stages [98]. Conclusions and inferences will be synthesized for a more contextualized and thorough understanding of the preliminary impact of the intervention. The mixed methods design will serve two purposes: (1) complementarity and (2) expansion [99,100]. Qualitative findings will be connected to quantitative findings where the former will provide explanations and context for findings produced by the latter. Moreover, qualitative findings will complement our understanding of attendance and participant satisfaction with the intervention.

## Results

The team completed the recruitment of phase 1 study participants (phase 1, aim 1) in January 2023. A total of 32 youths and 16 health care providers from 2 health clinics were recruited. Youths and health care providers participated in focus group discussions (4 groups for youths and 2 groups for health care providers). During the discussions, participants shared their feedback on the proposed Suubi-Mhealth intervention content, methods, design, and format, to inform the development of the app. All focus group discussions were audio-recorded to ensure accuracy of participants’ responses. Transcription and translation of focus group discussions have been completed. The team is currently conducting data analysis of participants’ responses, to inform the development of the Suubi-Mhealth app content. Concurrently, the development of Suubi-Mhealth content is ongoing. Specifically, the team is developing short videos to be incorporated into the app, including those related to depression and its symptoms, strategies to reduce negative thoughts, skill building, daily activities, and goal monitoring, to be incorporated into the app. Upon completion, the app will be tested among 30 youths to garner feedback on usability (phase 1, aim 2).

## Discussion

### Overview

People living with HIV, including adolescents, often struggle with mental health comorbidities that lower their adherence to ART treatment [7,9]. There is growing evidence that depression treatment may improve ART adherence and result in improved HIV outcomes [19,20]. This Suubi-Mhealth study will develop a mHealth intervention for use among Ugandan youths with comorbid HIV and depression, taking into account their unique contextual, cultural, and developmental needs. We expect Suubi-Mhealth to be an acceptable and feasible mHealth tool to reduce depression and improve ART adherence and overall mental health functioning among youths.

The study innovates in the following ways. First, while mHealth interventions have been tested and implemented to improve adherence to ART among youths [50,51,53], digital intervention strategies addressing depression plus ART adherence among youths living with HIV in SSA are very limited [101]. This study will generate data-driven knowledge to address depression and ART adherence and will ultimately improve HIV-related health outcomes for youths, including adherence to medication. Second, delivering CBT—a theoretically driven intervention, delivered via a mobile app—has the potential to facilitate timely linkage and access to mental health services for youths, one of the major challenges to addressing mental health in impoverished communities. Previous studies have documented the potential of CBT to improve ART treatment outcomes [102]. Third, the study makes use of existing local institutions—both research and community-based institutions and locally recruited and trained research staff—to deliver its intervention, building research capacity and ensuring eventual scale-up and sustainability. Fourth, partnering with local institutions, including health clinics and community organizations, as well as the youths, grounds the project with a practical understanding of the needs of youths in the greater Masaka region—an area of Uganda hardest hit by HIV or AIDS [4]. Finally, the study takes advantage of the increasing penetration of cellphones and mobile phone ownership in Uganda [46], to test and deliver an innovative, culturally-tailored intervention with significant public health outcomes. Taken together, this study will lay important groundwork for several initiatives at the intersection of digital therapeutics, HIV treatment, and mental illness, especially among SSA youths, as they transition through adolescence and into adult HIV care settings.

### Dissemination

The research team will facilitate learning across stakeholders and maximize the use of the evidence generated through dissemination meetings. Uganda’s HIV prevention and treatment guidelines and policies recognize the burden and impact of mental health and HIV stigma on HIV treatment outcomes among youths, their families, and communities. If findings warrant, we will leverage these policy guidelines to maximize dissemination of study findings.

### Data Sharing

The research team is committed to open, timely, and widespread sharing and dissemination of study findings. The team adheres to the National Institutes of Health’s Public Access Policy that requires final, peer-reviewed papers to be submitted to the National Library of Medicine’s PubMed Central upon acceptance for publication. We will also make study results available via papers written for professional and layperson publications, presentations at scientific and professional conferences, special lectures or talks in academic and professional settings, websites, and newsletters. Once all data have been deidentified, cleaned, and validated, and the main findings have been published, the investigators expect to share data with the scientific community. The research team will make data sets available to any individual who makes a direct request to the principal investigators and indicates the data will be used for the purposes of research. In sharing participant data, the team will follow Office of Sponsored Projects’ data sharing agreements at Washington University in St. Louis, which are similar in their specification of the following conditions to be met before data are shared: (1) a formal research question is specified a priori; (2) names, affiliations, and roles of any other individuals who will access the shared data; (3) the deliverables—for example, paper, conference presentation—are specified a priori; (4) proper credit and attribution—for example, authorship, coauthorship, and order—for each deliverable are specified a priori; (5) a statement indicating an understanding that the data cannot be further shared with any additional individuals or parties without the Principal Investigators’ permission; and (6) IRB approval for use of the data (or documentation that IRB has determined the research is exempt).

The research team will strip data of identifiers that would permit linkages to individual research participants and variables that could lead to deductive disclosure of the identity of individual participants. We will share data in electronic format native to the software used by the research team; requestors are expected to handle converting electronic formats. Upon completion of the deliverables, the MPIs will instruct the requestor to destroy all copies of the data. If deliverables have not been produced yet, the agreement to share data will be revisited annually by the MPIs and the research team to decide whether to continue sharing or terminate the sharing agreement.

## Appendix

Multimedia Appendix 1 Peer-review reports from the National Institute of Mental Health (NIMH) | National Institutes of Health (NIH).

## Abbreviations

ART: antiretroviral therapy
CBT: cognitive behavioral therapy
ICHAD: International Center for Child Health and Development
IRB: institutional review board
mHealth: mobile health
PHQ-9: Patient Health Questionnaire-9
PRISM: Practical, Robust Implementation, and Sustainability Model
SSA: Sub-Saharan Africa
USE: Usefulness, Satisfaction, and Ease of Use
