# Supplementary material for: An mHealth Intervention to Address Depression and Improve Antiretroviral Therapy Adherence Among Youths Living With HIV in Uganda: Protocol for a Pilot Randomized Controlled Trial
Source: JMIR Res Protoc. 2024 Mar 8;13:e54635. doi: 10.2196/54635 (PMC10960218; doi:10.2196/54635)
Supplement: Multimedia Appendix 1 [file resprot_v13i1e54635_app1.pdf]

SUMMARY STATEMENT
(Privileged Communication)

PROGRAM CONTACT:
Leonardo Cubillos
leonardo.cubillos@nih.gov

Release Date: 03/29/2022
Revised Date:

Application Number: 1 R21 MH131044-01

Principal Investigators (Listed Alphabetically):

CAVAZOS-REHG, PATRICIA A
MUGISHA, JAMES
NABUNYA, PROSCOVIA (Contact)

Applicant Organization: WASHINGTON UNIVERSITY

Review Group: ZRG1 HDM-J (56)
Center for Scientific Review Special Emphasis Panel
Mobile Health: Technology and Outcomes in Low and Middle Income Countries (R21/R33 - Clinical Trial Optional)
AIDS - EXP. REV.

Meeting Date: 03/11/2022
Council: MAY 2022
Requested Start: 07/01/2022

RFA/PA: PAR21-303
PCC: 9G-GMH4
Dual PCC: MHEALTH
Dual IC(s): TW

Project Title: Suubi-Mhealth: A mobile health intervention to address depression and improve ART adherence among Youth living with HIV (YLHIV) in Uganda
SRG Action: Impact Score:26 Percentile:9 #
Next Steps: Visit https://grants.nih.gov/grants/next_steps.htm
Human Subjects: 30-Human subjects involved - Certified, no SRG concerns
Animal Subjects: 10-No live vertebrate animals involved for competing appl.
Gender: 1A-Both genders, scientifically acceptable
Minority: 5A-Only foreign subjects, scientifically acceptable
Age: 2A-Only Children, scientifically acceptable

Project Year	Direct Costs Requested	Estimated Total Cost
1	125,000	176,629
2	125,000	176,629
3	200,000	282,607
4	200,000	282,607
5	200,000	282,607
TOTAL	850,000	1,201,078

1R21MH131044-01 Nabunya, Proscovia

RESUME AND SUMMARY OF DISCUSSION: This R21/33 project develops a mobile app to improve antiretroviral therapy adherence among Ugandan youth with comorbid HIV and depression. Depression reduces adherence and treatment for depression is rare, particularly among adolescents. A mobile app that delivers cognitive behavioral therapy and social support could both improve mental health and reduce HIV transmission in this high-risk group. The research problem is important. Rigorous prior research and preliminary data are reviewed. A theoretical framework is proposed. There are strong letters of support. Capacity-building prospects are evident. Significance was deemed high. The superb investigative team brings prior collaboration, past and current NIH funding and complementary HIV and adolescent, mental and digital health expertise. The International Center for Child Health and Development at Washington University, Makerere University and Reach the Youth Uganda provide an excellent research environment. A mobile app and online platform that delivers cognitive behavioral therapy to reinforce HIV medication adherence and to treat depression among Ugandan youth living with HIV, allows them to socially network with therapists and peers, and links them to local mental health services, is innovative. Formative intervention development, feasibility and acceptability testing and impact measurement methods are outlined. Milestones are measurable. Potential challenges and workarounds are discussed. Mixed methods, use of existing instruments and mixed effects modeling add to scientific rigor. In discussion, several remediable weaknesses were identified. How the previous experience with substance use disorders will be used to adapt the digital tool for comorbid HIV and depression is not well explained. Outcomes to be measured do not map clearly onto the study aims. It is unclear whether focus groups will be gender-specific. How the discussion forum will be moderated and risks in providing mobile phones to the control group are not fully addressed. Caretakers and parents should also be studied. More information is needed on the local health clinics and partners who will treat the subjects- their characteristics, how they were selected and how well they perform. On balance, strengths quite exceeded weaknesses. The project will significantly impact mental health and HIV comorbidity among Ugandan adolescents, concluded the panel.

DESCRIPTION (provided by applicant): Approximately 1.7 million children under 15 years old were living with HIV in 2020; and most new HIV infections (85%) occurred in Sub-Saharan Africa (SSA). People living with HIV (PLHIV) often struggle with mental health comorbidities that lower their antiretroviral therapy (ART) adherence. However, 76% to 85% of PLHIV in SSA receive no treatment for serious mental health disorders, especially depression. Depression reduces ART adherence, which negatively impacts health and increases HIV transmission risks. Older adolescents (≥14 years) living with HIV are particularly vulnerable to these risks as caregivers withdraw or lessen their support during their transition to young adulthood. Moreover, older adolescents are also moving into larger and less accommodating adult HIV clinic settings and are at risk for dropping out of ART programs. Given that mental health services are severely under equipped in SSA, including in Uganda, and are inaccessible by many YLHIV, new solutions to increase access to mental health care and close the treatment gap are urgently needed. The overall goal of this proposed R21/33 study is to develop an mHealth intervention (Suubi-mhealth) for use among Ugandan youth (14-17 years) with comorbid HIV and depression, taking into account their unique contextual, cultural, and developmental needs. This digital therapy intervention delivered via a mobile application, will utilize the core tenets of cognitive-behavioral therapy (CBT) found to improve depression and ART adherence. The proposed study will specifically: Phase 1. R21 Aim 1: Develop and iteratively refine an intervention protocol for Suubi-Mhealth based on formative work to understand needs of youth living with HIV (YLHIV). We will conduct four focus groups with youth and two focus groups with health care providers (6-8 youth each) for feedback on intervention content and methods to increase participation and retention. R21 Aim 2: Based on results of Aim 1, explore the feasibility and acceptability subsequent refinement for the larger R33 phase. Phase of Suubi-Mhealth on a small scale (N=30), to inform 2. R33 Aim 1: Test the preliminary impact of

Suubi- Mhealth versus a waitlist control group (N=200), on youth outcomes (depression, ART adherence, mental health functioning, quality of life, stigma). R33 Aim 2: Examine barriers and facilitators for integrating Suubi-Mhealth into health care settings for YLHIV. The study will be conducted in 10 health clinics in the greater Masaka region in Southern Uganda. We expect for Suubi-Mhealth to be an acceptable and feasible mHealth tool to reduce depression, improve ART adherence and overall mental health functioning among YLHIV. If the results of this pilot are promising, then the next step is an R01 to rigorously test Suubi-Mhealth in a larger trial, spanning multiple sites across Uganda.

PUBLIC HEALTH RELEVANCE: Aligned with NIH's priority of supporting the development of innovative mobile health applications to improve clinical and public health outcomes and strengthen research capacity in low- and middle-income countries, the proposed Suubi-Mhealth study seeks to develop an mhealth intervention for use among Ugandan youth (14-17 years) with comorbid HIV and depression, taking into account their unique contextual, cultural, and developmental needs. This digital therapy intervention delivered via a mobile application, will utilize the core tenets of cognitive-behavioral therapy (CBT) found to improve depression and ART adherence. We expect for Suubi-Mhealth to be an acceptable and feasible mhealth tool to reduce depression, improve ART adherence and overall mental health functioning among youth living with HIV.

CRITIQUE 1

Significance: 4
Investigator(s): 2
Innovation: 4
Approach: 5
Environment: 1

Overall Impact: This is an R21/R33 grant application that proposes to develop, refine, and pilot test a mHealth intervention that delivers cognitive behavior therapy (CBT) that will be tailored for youth living with HIV (YLHIV) in the greater Masaka region, an area of Uganda with a 12% prevalence of HIV/AIDS. The target population is YLHIV aged 14-17 years who are receiving care at two HIV clinics (Phase 1) and ten HIV clinics (Phase 2). For Phase 1 (R21), the aims are to: 1) develop and iteratively refine an intervention protocol using information gathered from four focus groups of depressed YLHIV (n=6-8) and two health care provider focus groups; and 2) conduct a small pilot test (n=30) of depressed YLHIV to explore feasibility and acceptability as well as further refine the intervention. Phase 2 (R33) includes: 1) a randomized clinical trial (intervention vs. wait list control) of 200 YLHIV from 10 clinics that will be randomized at the clinic level (n=5) to intervention vs. waitlist control (n=100 per condition); and 2) semi- structured interviews with 30 purposively selected youth and research staff (n=10) to qualitatively examine barriers and facilitators. The significance of the research is supported by relatively high HIV/AIDS prevalence rates in Uganda, estimated 63% prevalence of depression in PLHIV, prior research supporting that treatment of depression may improve antiretroviral treatment (ART) adherence, knowledge gap for adolescents in Sub-Saharan Africa, and prior research supporting efficacy of CBT to reduce depressive symptoms and self-stigma related to having a mental illness. Feasibility of a mHealth intervention is supported by more than 70% of Ugandans owning a mobile phone. The guiding theoretical frameworks are the MHealth Development and Evaluation Framework, and PRISM (Practical, Robust Implementation, and Sustainability model). The main argument for the innovation is addressing the absence of mHealth interventions that include strategies to address depression plus ART adherence in this target population and leveraging existing partnerships

across the research and community-based institutions. The investigative team brings together expertise in public health, implementation science, mHealth, interventions research, research methodology, research capacity building, mental health and HIV prevention and treatment. Across investigators, there is evidence of prior and current successful collaborations. There is strong potential to further strengthen research capacity in this region of Uganda. The milestones to track progress are specific and measurable. Areas for potential improvement are predominantly related to the approach. Some of the main concerns were lack of information about the 10 clinics (clinic volume, # served in study age range, demographics, any community characteristics of clients served, staffing), lack of detail on how health care providers would be purposively sampled for focus groups, some lack of consistency between theoretical model, data summary (Table 3), and data analysis.

1. Significance:

Strengths

- The argument for focusing on this target population is strong: high prevalence of HIV in Uganda and growing population of YLHIV due to improvement in access and use of HIV services.
- Rationale for targeting depression as primary outcome is concisely described and well cited.
- Feasibility of a mHealth intervention is strongly supported by 70% of Ugandans own a mobile phone. Four prior studies are also cited that are specific to successful use of MHealth technologies to deliver health interventions relevant to this research.

Weaknesses

- B.4 appears to be supporting focusing the study on youth that are transitioning between adolescents and young adulthood, but this study focuses solely on legal minors and adolescents ranging from ages 14-17 years.
- An option could be to also mention that prevalence of depression rises during adolescence, especially among females.
- Reference is missing to support “costly long-term consequences” for not addressing mental health needs of YLHIV.
- The summary statement related to how interventions should take into account numerous treatment barriers does not fit well under the heading “Cognitive behavior therapy (CBT) is a promising approach to reduce depression and improve ART adherence.
- It might be premature to conclude that the proposed intervention is a “sustainable approach.”

2. Investigator(s):

Strengths

- The majority of the investigative team is based at Washington University in St. Louis, and together they have strong track record of collaboration. For example, Drs. Nabunya and Ssewamala have collaboration on five NIH R01’s, two R21’s and one R34.
- Dr. Mugisha (country MPI) has expertise in social work and brings more than 20 years of clinical research experience including focus group facilitate with youth in Uganda who had depression and HIV. She also collaborates with Dr. Nabunya (Lead PI) on her R21 that utilizes group CBT and multiple family group approaches to address HIV-related stigma among adolescent living with HIV and caregivers in Uganda.

- Together, the team also currently implements several NIH-funded training programs to increase HIV and mental health research capacity in the regions and a collaborative hub grant.
- The roles of the MPI's and PD Leadership Plan are sound.

Weaknesses

- Dr. Nabunya is a Research Assistant Professor, but will work in close collaboration with Dr. Cavazos (MPI) who is Professor of Psychiatry.

3. Innovation:
Strengths

- The multi-prong strategy of developing an intervention for addressing both depression and ART adherence in this target population is novel.
- Although not specifically stated, the proposed intervention has the potential to shift the clinical paradigm from traditional clinic-based care to more patient-centered care using Mhealth for YLHIV teens who are depressed.
- There may be potential to identify youth at risk for depression early during their ART care and mitigate more mild depression using CBT early in their course of depression.

Weaknesses

- There was some redundancy with the argument for the significance of the study
- The statement that delivering CBT by mobile app will "facilitate timely linkage and access to mental health services for YLHIV" might be true, but this study does not appear to be examining these service use outcomes.
- The leveraging of existing partnerships and further building research capacity might fit better under significance.

4. Approach:
Strengths

- The transition milestones are specific, quantifiable and allocated project months are reasonable.
- Eligibility criteria for R21 Aim 1 are clearly described.
- Focus groups will be conducted in Luganda under the leadership of the country MPI, Dr. Mugisha, who has extensive experience conducing focus groups
- Inductive thematic analysis of focus group transcripts is clearly described.
- Decision rules to continue to full RCT are operationally defined within the constructs of feasibility, acceptability, and preliminary impact (efficacy?).
- Purposeful sample of high and low app users (which is operationally defined) will be used for the implementation evaluation.

Weaknesses

- During Phase 1 (Aim 1), are males and females in separate focus groups?
- During this phase, are youth purposively selected to enhance a breadth of experiences/perspectives to assess need?
- What was the rationale for not including a parent/primary caregiver/family member perspectives at this early stage?

- Please specify how health care providers would be purposively sampled to enhance breath of perspectives (disciplines, roles, experience providing care to target population and their families) to better inform intervention content and delivery, subject recruitment at the clinic sites, implementation of the small pilot, etc.
- For the quantitative measures for the beta test (R21, Aim 2) the study variables for engagement with the app were good, but I assume the data are also time stamped so there could be an opportunity to explore how these vary over time during the 2 months? Do users engage early and a lot and then stop early? If they engage early, are they more likely to use the app more continuously over time? When is the qualitative interview conducted? 2 months following index start date of being given the phone or first use?
- Although noble, it may not be feasible to integrate “all participants’ feedback” into the app. There might be issues of feasibility, costs and maybe differences of opinions that might have to be weighed when further refining the intervention.
- There is little information provided about the pool of 10 clinics (or 2 clinics for Phase 1). Any insights in clinic volume, # served/year, demographics of patients served, any differences in clinical severity, community served by clinic, staffing, how HIV care might differ across clinics?
- Please improve consistency in how randomization is described. It appears that the 10 clinics will be randomized based on size (total # youth served) and that the health facility level varies (level II, II, IV or hospital). Was the team envisioning a stratified random sample? Is clinical severity confounded by health facility level? In E.6.4 the text implies randomization at the individual level within a clinic following informed consent. Under sample size/power it states n=5 clinics per condition.
- It appears that the intervention will also include a social networking center. Will use of this feature be include in the quantitative data? Same issue re: rewards feature.
- The intervention ends at 2 months. In Table 3 it appears the 6-month time point is reported to align with “3 months post-intervention”. If you measure in weeks following index start date of intervention (defined as?), then the time intervals are baseline, 4 weeks, 8 weeks and if you want to assess 12 weeks after end of intervention, this would be at 20 weeks? Or, do you mean the 6-month time point aligns with 4 months post- intervention?
- There is some lack of cohesion across theoretical modal, data summary (Table 3), and data analysis. Proposed mediators are not identified in the table. A figure that summarized the main study variables by primary and secondary outcomes, mediators, moderators, covariates that more closely aligned between Table 3 and data analysis plan would be helpful.
- Some of the study variables (i.e., psychologic distress, social supports) are not specified in the list of variables in the aim.

5. Environment:

Strengths

- The health clinics included in this study are currently collaborating with the International Center for Child Health and Development (ICHAD) and the Reach the Youth Uganda (RTY) in study region and on several other studies.
- The research capacity of the in-country collaborators and partners in Uganda is likely to be strengthened because the ICHAD will house the proposed study in Uganda, the RTY is the implementation partners and Makerere University includes research partners.

Weaknesses

- None

Milestones (Reference the language in Section V: Additional Review Criteria of PAR 21-303):
Strengths

- Milestones were clearly defined and quantifiable

Weaknesses

- None

Study Timeline:
Strengths

- The time intervals for each milestone were reasonable.
- R21 and R33 tasks seems feasible within time allotted.

Weaknesses

- None

Protections for Human Subjects:
Acceptable Risks and/or Adequate Protections

- No concerns. Protocols to respond to possible risk included

Data and Safety Monitoring Plan (Applicable for Clinical Trials Only):
Acceptable

- Clearly described

Inclusion Plans:

- Sex/Gender: Distribution justified scientifically
- Race/Ethnicity: Distribution justified scientifically
- For NIH-Defined Phase III trials, Plans for valid design and analysis: Not applicable
- Inclusion/Exclusion Based on Age: Distribution justified scientifically
- No concerns

Vertebrate Animals:
Not Applicable (No Vertebrate Animals)

Biohazards:
Not Applicable (No Biohazards)

Applications from Foreign Organizations:
Justified

- High prevalence of HIV and depression in target population. MHealth intervention has

potential to improve care among this underserved group. Strong potential to further build research capacity in this area of Uganda.

Budget and Period of Support:
Recommend as Requested

CRITIQUE 2

Significance: 2
Investigator(s): 1
Innovation: 2
Approach: 3
Environment: 1

Overall Impact: This application aims to develop a mobile health intervention, Suubi-Mhealth, for Ugandan youth with HIV and depression. The investigators provide highly detailed preliminary data on the need for this intervention and the conceptual frameworks underlying their approach. The R21 phase would develop an intervention protocol for Suubi-Mhealth based on focus groups, followed by a feasibility study in 30 YLHIV; the R33 phase would test preliminary impact of the intervention in 200 participants ages 14-17 years from 10 health clinics in a parallel-group pilot RCT – with the control arm being a waitlist condition – to examine effects on ART adherence, depression/mental health symptoms, QOL, and engagement with the app over 2 months, with 6 month follow up to examine persistence of effects. The team would also qualitatively examine barriers and facilitators for the intervention. Overall it is a very clearly written application with only a few minor methodological issues, such as questions about the method of randomization, and how the social network component of the intervention will be moderated. The investigators are highly experienced in intervention development and have a track record of successful collaboration between Washington University and academics/research teams in Uganda.

1. Significance:

Strengths

- The application is extremely well-written and organized. Strong arguments are provided for the need to address depression in YLHIV, gaps in research, and mechanisms (e.g., the fact that youth who are aging out of pediatric services may drop out of ART programs.) The investigators clearly explain why their approach could improve HIV outcomes through ART adherence and lower transmission.
- It is clear from the Significance section that the research team understands the complexity of the context of this intervention and how it would need to be adapted to account for treatment barriers and multi-level factors.
- PRISM and mHealth Development and Evaluation frameworks will be followed, which increases the rigor of their approach

Weaknesses

- In the Preliminary Studies, it is helpful to see Dr. Cavazos' preliminary work on a digital therapeutic tool, but it would help to hear consideration of the possible limitations of applying

these insights into a treatment for depression (not substance abuse) within a different population/geographical location.

2. Investigator(s):

Strengths

- PI Nabunya has extensive expertise in HIV-stigma reduction interventions; family and community-based support systems as protective factors for the development and wellbeing of children and youth in low resource settings
- MPI Cavazos has expertise in addiction, mental health, social media, and interventions delivered via mobile apps
- The research team is extremely experienced and has collaborated successfully in the past.
- Co-I Mugisha will oversee cultural adaptation of the intervention
- Good that they are using a private industry team rather than trying to get academics/freelancers to work on the technical features.

Weaknesses

- None noted

3. Innovation:

Strengths

- The proposed intervention fills gaps in treatment of depression in YLHIV
- The intervention would also build research capacity and sustainability over time
- Clear attention is paid to the practical, local experiences of individuals in the Masaka region

Weaknesses

- It would be interesting to know more about how the investigators see their economic empowerment intervention intersecting with CBT/mHealth

4. Approach:

Strengths

- Possible content/framework for Suubi-Mhealth modules is clearly described
- R21 Phase – inclusion criteria, recruitment methods, screening, and rationale for these decisions is clearly described, and reflects the study team’s existing expertise carrying out research in these clinical settings.
- Focus groups not only ask about impressions of expert-suggested content and design, but also about their own suggested components and how to retain participants over time (which is an important problem to address with MHealth interventions, once the initial novelty has worn off).
- R21 phase uses rigorous/standardized methods for evaluating usability, a variety of engagement metrics, and will be followed by adaptation based on feedback that is clearly described.
- R33 assessments and timeframes are clearly described

- Moderation analyses by engagement with the app and sex are important to include, as are mediation analysis to examine mechanism of action (i.e., whether depression symptoms mediate ART adherence).

Weaknesses

- Rationale for the social networking center is provided, but there is insufficient attention to the risks and benefits of social media interactions on teen mental health. What steps (e.g., forum moderation) will be taken to ensure interactions do not turn negative, judgmental, or unintentionally harmful (e.g., promoting self-harm, which has occurred on other social networks)?
- In the R33, it is unclear whether the randomization will be at the clinic level, which is stated several times in the application – but in section E.6.4 it seems it will be done at the individual level?
- In the R33 phase, the control group will be provided with a smartphone as well, and the researchers may want to consider what mental health effects (positive or negative) might occur with regular access to social media, news, or ability to contact others. Perhaps this could be explored in the post-intervention participant interviews?

5. Environment:

Strengths

- ICHAD at Washington Univ has a strong track record of carrying out intervention studies in SSA, coordinating with a team at ICHAD Uganda.
- Involvement of the SMART Africa center (transdisciplinary research center aimed at reducing child mental health service and research gaps in Ghana, Kenya and Uganda) and Makerere University will allow sustainability of research efforts
- Reach the Youth has collaborated with ICHAD on several grants in the past

Weaknesses

- None noted

Milestones (Reference the language in Section V: Additional Review Criteria of PAR 21-303):

Strengths

- Thorough consideration of the different steps that will be taken throughout the R21 and R33 phases of the project
- Clear decision rules are provided for continuing to full RCT

Weaknesses

- Milestones in the Timeline document are listed as essentially the components of the grant, not benchmarks for how the team will know that they have completed them.

Study Timeline:

Strengths

- Very specific timeline and steps of intervention development/testing are provided.

Weaknesses

- None noted

Protections for Human Subjects:
Acceptable Risks and/or Adequate Protections

- Very thorough

Data and Safety Monitoring Plan (Applicable for Clinical Trials Only):
Acceptable

- Appropriate attention to mental health risks/suicidality

Inclusion Plans:

- Sex/Gender: Distribution justified scientifically
- Race/Ethnicity: Distribution justified scientifically
- For NIH-Defined Phase III trials, Plans for valid design and analysis: Not applicable
- Inclusion/Exclusion Based on Age: Distribution justified scientifically
- No concerns

Vertebrate Animals:
Not Applicable (No Vertebrate Animals)

Biohazards:
Not Applicable (No Biohazards)

Applications from Foreign Organizations:
Justified

- Proposal examines depression in YLHIV in Uganda

Budget and Period of Support
Recommend as Requested

CRITIQUE 3

Significance: 1
Investigator(s): 1
Innovation: 1
Approach: 1
Environment: 1

Overall Impact: This R21/R33 application seeks to develop an mhealth intervention to address depression among young adults living with HIV (YLHIV) in Uganda. The researchers make a compelling case for the public health impact of depression on ART adherence among YLHIV. The research is proposed by an exceptional group of investigators collaborating

1. Significance:

Strengths

- A compelling case for the impact of depression on ART adherence
- Compelling evidence is made for not only health disparities related to HIV and depression but treatment inequities including limited access

Weaknesses

- None noted by reviewer.

2. Investigator(s):

Strengths

- The PI (of MPI) is clearly dedicated to addressing health disparities and treatment inequities among those living with HIV in Sub-Saharan Africa as evidenced by a long track record of funding and extensive publication
- Dr Mugisha has collarated with both Drs Nabunya and Ssewamala brings expertise in both HIV and suicide research. They also strengthen the connections between the University of Washington and the ICHAD given his location in Uganda
- Dr Cavazos-Rehg compliments the research team with significant clinical expertise as well as research in substance use and mental health
- The MPI plan (with Nubanya as contact PI and Cavazos-Rehg and Mugisha as co-leads) is well justified and clearly defined roles and procedures for resolving any potential conflicts
- Dr Filiatreau bring expertise in epidemiology of mental health and HIV in underserved populations
- Dr Ssewamala has over 100 publications in this research topic. As Director of International Center for Child Health and Development, he is well positioned to facilitate recruitment and other research activities in

Weaknesses

- Overall, the research team is exceptionally well qualified to conduct the proposed research

3. Innovation:

Strengths

- Overall, targeting depression as a risk factor associated with ART adherence among young adults living with HIV in Uganda is highly innovative.
- Leveraging CBT to inform the development of an mhealth solution is innovative
- Leveraging existing and productive international collaborations in Uganda is a unique strength of the application
- Adhering to both the mHealth Development and Evaluation Framework and PRISM is innovative

Weaknesses

- Overall, both the individual elements and the research as a whole are highly innovative

4. Approach:

Strengths

- Leveraging the mhealth development and evaluation framework is a significant strength
- Leveraging the PRISM (Practical, Robust, Implementation and Sustainably Model) is a significant strength
- Preliminary studies highly favorable
- Intervention based on CBT is very promising. Intervention is well articulated.
- Clear commitment to capacity building
- Accounted for impact of COVID on research efforts
- Clear evidence of recruitment capacity
- The hypotheses, measurement procedures and statistical analyses are very robust

Weaknesses

- It may be beneficial to interview parents as they may provide unique insights into potential risk and protective factors of depression in young adults living with HIV that could inform the development of the mHealth application (minor)

5. Environment:

Strengths

- Washington University in St Louis and its affiliation institutions are well prepared to successfully carry out the proposed research
- The International Center for Child Health and Development, with two locations in Uganda, is well positioned to ensure successful execution of the proposed research
- Letter of support express strong commitment to the scope of work and the overall success of the project

Weaknesses

- None noted

Milestones (Reference the language in Section V: Additional Review Criteria of PAR 21-303):

Strengths

- Clearly and appropriately defined as well as reasonable

Weaknesses

- None noted

Study Timeline:

Strengths

- Well laid out and highly feasible

Weaknesses

- None noted

Protections for Human Subjects:

Acceptable Risks and/or Adequate Protections

Data and Safety Monitoring Plan (Applicable for Clinical Trials Only):

Acceptable

- Procedures to protect human subjects are commiserate with the risk and benefits of the proposed study.

Inclusion Plans:

- Sex/Gender: Distribution justified scientifically
- Race/Ethnicity: Distribution justified scientifically
- For NIH-Defined Phase III trials, Plans for valid design and analysis:
- Inclusion/Exclusion Based on Age: Distribution justified scientifically
- Study will target young adults between the ages of 14 and 17

Vertebrate Animals:

Not Applicable (No Vertebrate Animals)

Biohazards:

Not Applicable (No Biohazards)

Applications from Foreign Organizations:

Justified

- Application leverages history of collaboration between the University of Washington and the International Center for Child Health and Development in Uganda per PAR

Resource Sharing Plans:

Acceptable

Budget and Period of Support:

Recommend as Requested

THE FOLLOWING SECTIONS WERE PREPARED BY THE SCIENTIFIC REVIEW OFFICER TO SUMMARIZE THE OUTCOME OF DISCUSSIONS OF THE REVIEW COMMITTEE, OR REVIEWERS' WRITTEN CRITIQUES, ON THE FOLLOWING ISSUES:

PROTECTION OF HUMAN SUBJECTS: ACCEPTABLE

INCLUSION OF WOMEN PLAN: ACCEPTABLE

INCLUSION OF MINORITIES PLAN: ACCEPTABLE

INCLUSION ACROSS THE LIFESPAN: ACCEPTABLE

COMMITTEE BUDGET RECOMMENDATIONS: The budget was recommended as requested.

Footnotes for 1 R21 MH131044-01; PI Name: Nabunya, Proscovia

Ad hoc or special section application percentiled against "Total CSR" base.

NIH has modified its policy regarding the receipt of resubmissions (amended applications).See Guide Notice NOT-OD-18-197 at https://grants.nih.gov/grants/guide/notice-files/NOT-OD-18-197.html. The impact/priority score is calculated after discussion of an application by averaging the overall scores (1-9) given by all voting reviewers on the committee and multiplying by 10. The criterion scores are submitted prior to the meeting by the individual reviewers assigned to an application, and are not discussed specifically at the review meeting or calculated into the overall impact score. Some applications also receive a percentile ranking. For details on the review process, see http://grants.nih.gov/grants/peer_review_process.htm#scoring.

MEETING ROSTER

Center for Scientific Review Special Emphasis Panel
CENTER FOR SCIENTIFIC REVIEW
Mobile Health: Technology and Outcomes in Low and Middle Income Countries (R21/R33 - Clinical Trial Optional)
ZRG1 HDM-J (56)
03/11/2022

Notice of NIH Policy to All Applicants: Meeting rosters are provided for information purposes only. Applicant investigators and institutional officials must not communicate directly with study section members about an application before or after the review. Failure to observe this policy will create a serious breach of integrity in the peer review process, and may lead to actions outlined in NOT-OD-14-073 at https://grants.nih.gov/grants/guide/notice-files/NOT-OD-14-073.html, NOT-OD-15-106 at https://grants.nih.gov/grants/guide/notice-files/NOT-OD-15-106.html, and NOT-OD-18-115 at https://grants.nih.gov/grants/guide/notice-files/NOT-OD-18-115.html, including removal of the application from immediate review.

CHAIRPERSON(S)

FIELD, CRAIG A, PHD, MPH
PROFESSOR
DEPARTMENT OF PSYCHOLOGY
COLLEGE OF LIBERAL ARTS
THE UNIVERSITY OF TEXAS AT EL PASO
EL PASO, TX 79912

MEMBERS

BANTUM, ERIN O, PHD
ASSOCIATE PROFESSOR
DEPARTMENT OF CANCER PREVENTION AND CONTROL
UNIVERSITY OF HAWAI'I CANCER CENTER
HONOLULU, HI 96813

GLASS, NANCY E, PHD
PROFESSOR
CENTER FOR GLOBAL HEALTH
SCHOOL OF NURSING
JOHNS HOPKINS UNIVERSITY
BALTIMORE, MD 21205

GRAETZ, ILANA, PHD
ASSOCIATE PROFESSOR
DEPARTMENT OF HEALTH POLICY AND MANAGEMENT
ROLLINS SCHOOL OF PUBLIC HEALTH
EMORY UNIVERSITY
ATLANTA, GA 30322

HAWK, MARY E, BS, DRPH, MSW
ASSOCIATE PROFESSOR
BEHAVIORAL AND COMMUNITY HEALTH SCIENCES
SCHOOL OF PUBLIC HEALTH
UNIVERSITY OF PITTSBURGH
PITTSBURGH, PA 15261

HERSH, WILLIAM R, BS, MD
PROFESSOR
DEPARTMENT OF MEDICAL INFORMATICS
AND CLINICAL EPIDEMIOLOGY
SCHOOL OF MEDICINE
OREGON HEALTH & SCIENCE UNIVERSITY
PORTLAND, OR 97239

HOCHHEISER, HARRY S, BS, MS, PHD
ASSOCIATE PROFESSOR
DEPARTMENT OF BIOMEDICAL INFORMATICS
INTELLIGENT SYSTEMS PROGRAM
SCHOOL OF COMPUTATION AND INFORMATION
UNIVERSITY OF PITTSBURGH
PITTSBURGH, PA 15206

JOHNSON, CONSTANCE M, PHD, MS, BSN
PROFESSOR
SCHOOL OF NURSING
UNIVERSITY OF TEXAS
HOUSTON, TX 77030

RADESKY, JENNY S, MD
ASSISTANT PROFESSOR
DEPARTMENT OF DEVELOPMENTAL BEHAVIORAL
PEDIATRICS
UNIVERSITY OF MICHIGAN
ANN ARBOR, MI 48109

RUGGIERO, LAURIE, PHD
PROFESSOR
DEPARTMENT OF BEHAVIORAL HEALTH AND NUTRITION
COLLEGE OF HEALTH SCIENCES
UNIVERSITY OF DELAWARE
NEWARK, DE 19716

SCHOENBERGER, YU-MEI M, PHD, MPH, BS
ASSISTANT PROFESSOR
DIVISION OF PREVENTIVE MEDICINE
UNIVERSITY OF ALABAMA AT BIRMINGHAM
BIRMINGHAM, AL 35205

TOROUS, JOHN, BS, MD, MS, PSYCHIATRY
DIRECTOR
DIGITAL PSYCHIATRY DIVISON
BETH ISRAEL DEACONESS MEDICAL CENTER
HARVARD MEDICAL SCHOOL
BOSTON, MA 02115

ZIMA, BONNIE T, MD, MPH
PROFESSOR
DEPARTMENT OF PSYCHIATRY AND BEHAVIORAL SCIENCES
UNIVERSITY OF CALIFORNIA AT LOS ANGELES
LOS ANGELES, CA 90024

SCIENTIFIC REVIEW OFFICER

MCQUESTION, MICHAEL J, PHD
SCIENTIFIC REVIEW OFFICER
CENTER FOR SCIENTIFIC REVIEW
NATIONAL INSTITUTES OF HEALTH
BETHESDA, MD 20892

Consultants are required to absent themselves from the room during the review of any application if their presence would constitute or appear to constitute a conflict of interest.
